# Origin of irregular X-ray mirage fringes from a bent, thin crystal

**DOI:** 10.1107/S2053273322006143

**Published:** 2022-07-28

**Authors:** Tomoe Fukamachi, Takaaki Kawamura

**Affiliations:** a Saitama Institute of Technology, Fukaya, Saitama 369-0293, Japan; b CMC Center, Tokyo University of Technology, Hachioji, Tokyo 192-0982, Japan; c University of Yamanashi, Kofu, Yamanashi 400-8510, Japan; Deutsches Electronen-Synchrotron, Germany

**Keywords:** interference fringes, mirage fringes, bent crystal, dynamical theory of X-ray diffraction

## Abstract

The dynamical theory of diffraction is used to analyse irregular X-ray mirage interference fringes observed in Si220 X-ray reflection topography from a weakly bent, thin crystal due to gravity. The origin of the irregular fringes is attributed to the interference between mirage diffracted beams and a reflected beam from the back surface, which is a new type of interference fringe. The irregular fringes are reproduced by calculating the reflected intensities numerically. The effects of absorption and thermal vibration are quite important for the reproduction. The result shows that the interference fringes depend on the strain as well as the thickness of the crystal, which indicates that the fringes should be useful for analysing weak strain in a crystal as an application.

## Abstract 2

Irregular X-ray mirage interference fringes observed in Si220 X-ray reflection topography from a weakly bent, thin crystal are analysed using the dynamical theory of diffraction. The origin is the interference between two or more mirage diffracted beams and one reflected beam from the back surface.

## 1. Introduction

**Figure 1:**
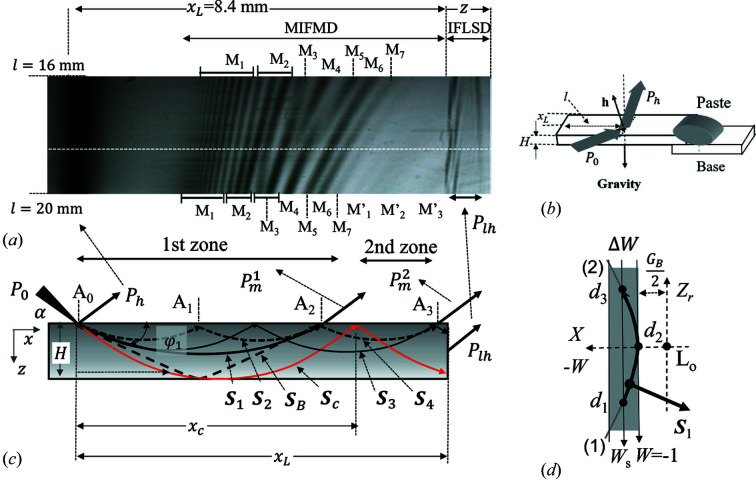
(*a*) Observed Si220 topography from a weakly bent plane-parallel crystal. The intensity profile in Fig. 6 ▸(*a*) is obtained along the horizontal dashed line. (*b*) Optical geometry around the crystal. *P*_0_ and *P_h_* represent the incident and the reflected beams, respectively. (*c*) Schematic illustration of the beam trajectories. 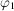 is the angle between *S*_1_ and the lattice plane at A_0_. (*d*) The real part of the dispersion surface (thick solid line). The abscissa represents the *X* axis and the ordinate the 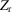 axis. L_0_ is the Lorentz point at ( 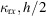 ). The part of the dispersion surface below *d*_2_ belongs to branch (1) and the part above *d*_2_ belongs to branch (2). The width of the shaded area corresponds to the divergent angle of the incident beam.

In a weakly bent crystal with a constant strain gradient, the index of refraction is variable with respect to the depth. When the X-ray beam enters the crystal, it propagates along a hyperbolic trajectory and is reflected back to the entrance surface as shown by 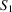 in Fig. 1 ▸, which is called mirage diffraction (Authier, 2001 ▸). Jongsukswat *et al.* (2012 ▸) have observed interference fringes of mirage diffracted beams (IFMD) from a weakly bent plane-parallel crystal and measured the strain of the crystal. The IFMD are caused by the interference between two mirage diffracted beams such as 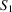 and 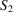 shown in Fig. 1 ▸(*c*). Fukamachi *et al.* (2011*b* ▸) have observed another type of mirage interference fringe caused by interference between diffracted beam 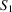 and reflected beam 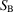 from the back surface (IFMRB) when the strain gradient of a thin crystal is very small. The fringe spacing of IFMD decreases as a function of the distance between the incident point and the exit point (*x*), whereas that of IFMRB increases. It is easy to distinguish IFMD and IFMRB. However, IFMD and IFMRB can coexist depending on the strain gradient and the thickness of the crystal. We report on irregular mirage interference fringes when IFMD and IFMRB coexist and their origin using the dynamical theory of diffraction.

## 2. Experimental results

Fig. 1 ▸(*a*) shows the observed Si220 topography, which is a part of Fig. 3 of Jongsukswat *et al.* (2013 ▸). The sample was a plane-parallel Si single crystal 50 mm long, 15 mm wide and 0.28 mm thick. One end of the sample was pasted to an aluminium base with pine resin as shown in Fig. 1 ▸(*b*). The sample was bent due to the gravity force. The diffraction experiments were carried out at the bending-magnet beamline BL-15C, Photon Factory, KEK, Tsukuba, Japan. The incident X-rays were σ polarized and monochromated by a Si(111) double-crystal monochromator. The X-ray energy was 11100 ± 0.5 eV. The beam size was 0.02 mm long and 4 mm wide. The details of the experiment are described by Jongsukswat *et al.* (2013 ▸).

Fig. 1 ▸(*a*) shows the topography in the range of distance (*l*) between 16 and 20 mm from the free edge of the sample. The dark contrast around 1 mm from the left is the primary diffraction. The fringe contrasts observed on the right side of it are denoted as MIFMD (modified IFMD) to be studied in this paper. Fig. 1 ▸(*c*) shows schematically the trajectories of the refracted beams in the crystal for formation of IFMD and IFMRB. Here the X-ray beam propagating along the energy flow, that is the Poynting vector of the beam, is referred to as the refracted beam. 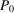 and 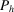 represent the incident beam at the point A_0_ and the primary diffracted beam, respectively. The refracted beam 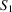 propagates along a hyperbolic trajectory and is emitted from the entrance surface at A_2_ without reaching the back surface – it is called the first-order mirage diffracted beam. Similarly, the beam 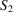 is another mirage diffracted beam reflected once from the entrance surface at A_1_, called the second-order mirage diffracted beam. The beam 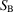 is reflected from the back surface and emitted from the entrance surface at A_2_, called the back-surface reflected beam. The beams 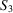 and 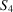 should be called the second- and third-order mirage diffracted beams emitted at A_3_, respectively. The longest distance 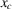 from the incident point to the emitted point for observing the first-order mirage diffracted beam is given by the mirage beam 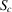 whose vertex of the trajectory is at the back surface. Interference of the first-order mirage diffracted beam with higher-order beams and the back-surface reflected beam 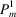 can be observed for 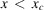 , which is called the first zone hereafter. Interference between mirage diffracted beams higher than the first order 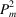 can be observed for 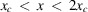 , which is called the second zone. In Fig. 1 ▸(*a*), the distance from the incident point to the lateral surface 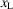 is smaller than 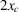 . The second zone is given by 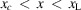 . The fringes on the rightmost side are the diffracted beams emitted from the lateral surface 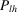 and are called IFLSD (interference fringes emitted from the lateral surface in the diffracted beam direction).

In the first zone, seven groups of fringes from M_1_ to M_7_ may be attributed to IFMD as will be shown in Section 4.2. As the crystal is bent due to gravity, the load is constant along the gravity. The strain gradient (β) is proportional to the square of the distance (*l*) from the free end according to rod theory. By using the procedure described by Jongsukswat *et al.* (2012 ▸), the strain gradients determined from the positions of the third peak (M_3_) of IFMD are β = 0.15 mm^−1^ at the upper end (*l* = 16 mm) and β = 0.23 mm^−1^ at the lower end (*l* = 20 mm) of Fig. 1 ▸(*a*).

In the first IFMD (M_1_), there are six fine interference fringes attributable to IFMRB. In the second IFMD (M_2_), there are three fine interference fringes attributable to IFMRB. The third fringe M_3_ shows dark contrast in the upper side and the contrast becomes darker in the lower side. The fringe M_4_ in the upper side disappears around *l* = 18 mm. The dark contrast M_4_ from the lower side disappears around the site (*l* ≃ 17 mm) in the middle of M_4_ and M_5_ coming from the upper side. The fringe M_6_ in the upper side is continuously connected to the fringe M_5_ in the lower side. In the second zone, three wide bands with low contrast can be seen on the lower side, which are labelled as M′_1_, M′_2_ and M′_3_. The behaviours of these interference fringes M_1_ to M_6_ as well as M′_1_ to M′_3_ are quite irregular.

## 3. Theoretical analysis

### 3.1. Basis

In the symmetric Bragg mode, the deviation parameter (*W*) from the Bragg condition of the angle 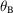 is given by

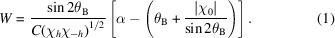

Here α is the incident glancing angle, *C* the polarization factor and 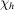 the *h*th Fourier coefficient of dielectric susceptibility of the crystal. According to Gronkowski & Malgrange (1984 ▸), the beam trajectory of the refracted beam in a weakly bent non-absorbing crystal is given by

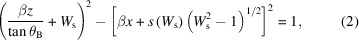

when the beam is incident outside the total reflection region ( 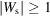 ). 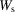 is the value of *W* at the incident point on the surface. The parameter 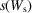 is 1 for 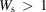 and −1 for 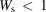 . The trajectory shows a hyperbolic form as seen in Fig. 1 ▸(*c*). The origin of the coordinate is taken at the incident point (A_0_), the *x* axis is along the direction from A_0_ to A_1_ and the *z* axis along the inward normal to the surface. The strain gradient parameter β is defined as

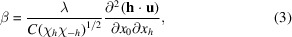

where 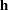 is the reciprocal vector, 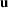 is the atomic displacement vector, λ the X-ray wavelength, 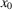 and 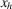 are the coordinates of the transmitted and diffracted beam directions, respectively.

When 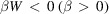 , a beam incident on the crystal at A_0_ propagates along the hyperbolic trajectory 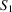 in Fig. 1 ▸(*c*) and changes the direction from +*z* to −*z* at the vertex ( 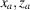 ) to reach the point A_2_. At A_2_, a part of the beam is emitted from the surface as the mirage diffracted beam and the rest is reflected back to the crystal. The reflected beam propagates along a similar hyperbolic trajectory starting from A_2_. This propagation process repeats. The electric field of the X-ray after *n* times of reflection is given by

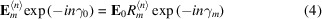

according to Fukamachi *et al.* (2010 ▸). Here 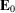 is the electric field of the incident X-ray, 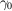 is the phase shift in vacuum and real. The phase shift of the refracted beam in the crystal 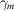 is complex by taking the absorption effect into account and given by

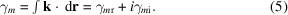

Here the integration is carried out along the trajectory, **k** is the wavevector in the crystal and **r** is the position vector. 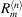 is given as

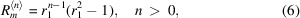

where 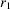 is expressed as

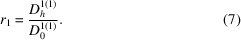




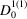
 and 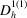 are the amplitudes of the 0 and **h**th Fourier coefficients of the electric displacement. The first number in the superscript represents the number of reflections and the second number (1) denotes the branch (1) of the dispersion surface.

We now need to calculate the phase shift 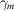 by using the dispersion surface. When the absorption is weak ( 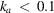 ), the complex dispersion surface for the Bragg mode is given as

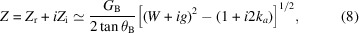

according to Fukamachi *et al.* (2002 ▸). 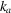 is expressed as

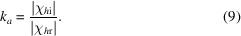




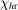
 and 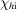 are the real and the imaginary parts of 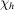 . 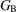 and *g* are given by

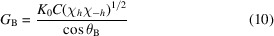

and

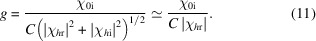




By squaring both sides of equation (8) and ignoring small terms 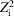 , 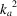 and 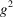 , equation (8) becomes

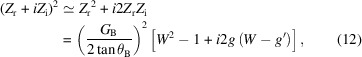

where the parameter 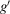 is given by

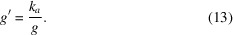




The real part of equation (12) becomes

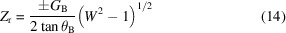

which agrees with the expression without absorption, indicating the validity of equation (2). The real part of the phase shift 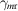 can be written as

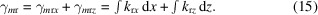

Here 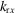 and 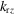 are *x* and *z* components of the real part 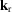 of the wavevector **k**


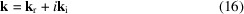

with 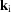 its imaginary part. As the coordinate of the Lorentz point is 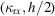 ( 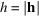 ) according to Fig. 1 ▸(*d*), 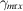 is rewritten as

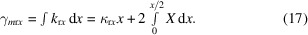




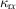
 is the real part of the average wavenumber in the crystal. The first term in equation (17) 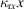 is the order of π, which is much smaller than the second term and can be neglected. As *X* in equation (17) is

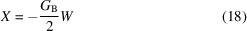

and

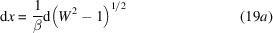




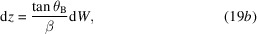

by using equation (2), the second term of equation (17) becomes

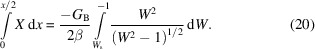




The phase shift 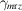 is obtained as

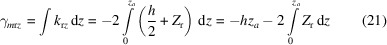

by referring to Fig. 1 ▸(*d*). The first term on the right-hand side 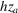 is the order of π and much smaller than the second term. As the second term is given by

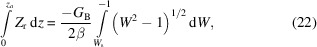

equation (15) becomes

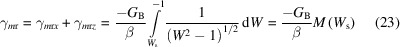

with

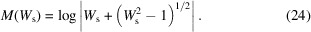




From the relation of the imaginary part of equation (12),

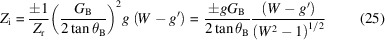

is obtained.

For the imaginary part of the phase shift 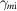 , only the *z* component needs to be considered. By using the relation

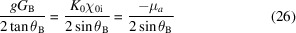

and equation (25), 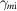 is given by

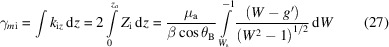




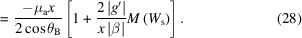

Here 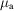 is the mean absorption coefficient. The exit point (*x*) of the mirage diffracted beam is given by

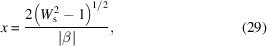

using the relation 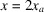 due to the symmetry of the trajectory. The initial value of the deviation parameter 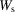 is obtained from the exit point (*x*) of the mirage diffracted beam. For a monatomic crystal with its atomic scattering factor being positive, the condition 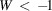 corresponds to the anomalous transmission of the Borrmann effect (Fukamachi *et al.*, 2002 ▸). In the following, we will study the fringes under this condition. As the X-rays are σ polarized in the experiment, the polarization factor *C* in equation (1) is 1.

### 3.2. Mirage interference fringes

In the first zone, the electric field of IFMD at A_2_ is given by

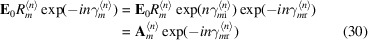

with 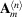 as

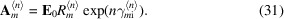




The electric field at A_2_ is written by

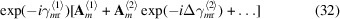

where the phase shift 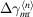 is defined by

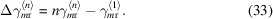

In the second zone, a similar equation is obtained without the first-order mirage diffracted beam.

IFMRB observed in the first zone has been explained by the interference between the first-order mirage diffracted beam 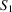 and a reflected beam from the back surface 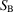 in Fig. 1 ▸(*c*) as expressed by

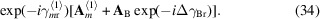

The amplitude ( 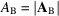 ) is given by

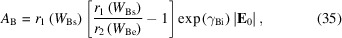

where 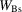 is the value of *W* of the beam 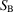 at the incident point and 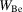 is that at the reflection point ( 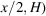 on the back surface. There is a relation between 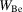 and 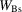 given by

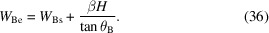




In equation (34), 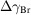 is given by

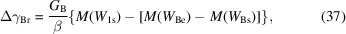

where 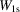 is the value of *W* at the incident point for the beam 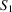 . 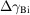 in equation (35) is given by

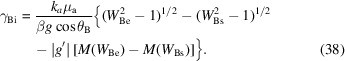




In order to explain irregular mirage interference fringes observed in the experiment, it is necessary to introduce MIFMD in the first zone given by adding a back-surface reflected beam to equation (32) as

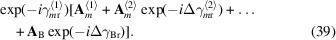

MIFMD in the second zone is composed of mirage diffracted beams higher than the first order, as the contribution of the back-surface reflected beam is small enough to be neglected.

### 3.3. Angular amplification

For observing MIFMD, it is necessary to have a certain width of Δ*W* to excite the first- as well as the higher-order mirage diffracted beams simultaneously. There is a relation

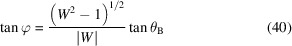

between *W* and φ which is the angle between the refracted beam and the lattice plane; 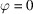 when 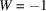 and 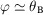 when 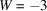 (Δ*W* = 2). If 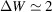 , the divergence angle of the refracted beam is approximately equal to 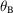 , the refracted beams are excited within the Borrmann triangle. The divergence angle of the incident X-rays corresponding to Δ*W* = 2 can be derived as Δα = 8.8 µrad by using equation (1). The angle amplification factor (Δφ/Δα) is approximately 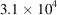 .

As the topography was taken by fixing the crystal as shown in Fig. 1 ▸(*c*), the incident glancing angle α was fixed. In order to observe mirage interference fringes, a finite divergence angle Δα is needed. The divergence angle Δα is related to the divergence angle 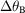 of the beam from the monochromator as

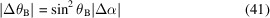

(Fukamachi *et al.*, 2014 ▸, 2015 ▸, 2019 ▸). By using the Bragg condition, the width Δ*E* of X-ray energy (*E*) and the angle width 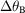 are related as

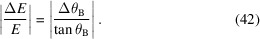

The refracted beams involved in the formation of mirage interference fringes have different wavelengths and different path lengths. Since the mirage interference fringes are observed in the experiment, the coherent condition is satisfied for the refracted beams. The details of the coherent condition have already been given in the previous papers by Fukamachi *et al.* (2014 ▸, 2015 ▸, 2019 ▸).

## 4. Results of calculation

### 4.1. Effects of absorption and thermal vibration

**Figure 2:**
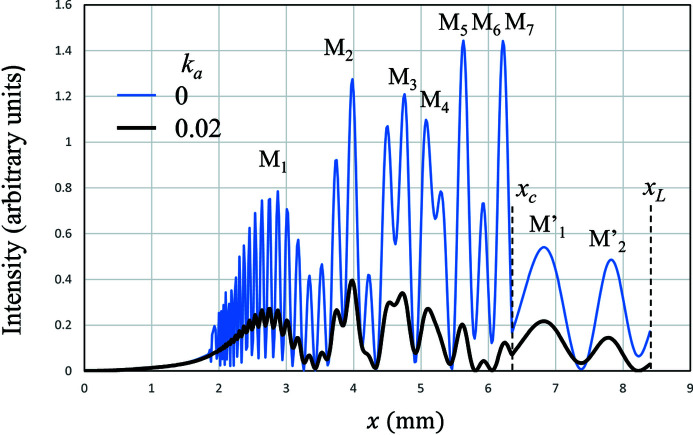
The calculated Si220 reflected intensities of MIFMD for 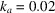 (in black) and 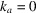 (in blue) in the case of β = 0.20 mm^−1^, 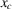 = 6.3 mm and 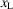 = 8.4 mm.

Fig. 2 ▸ shows the calculated results of MIFMD composed of mirage diffracted beams and a back-surface reflected beam. The used values of the strain gradient β and the parameter 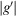 are 0.2 mm^−1^ and 1.0, respectively. In the first zone ( 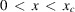 ), the interference fringes are composed of mirage diffracted beams from the first to the tenth order and one back-surface reflected beam. In the second zone ( 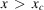 ), the interference fringes are composed of mirage diffracted beams from the second to the tenth order.

The effect of the mean absorption 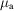 is shown in Fig. 2 ▸. According to equation (26), we have

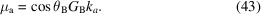

When the absorption is ignored, 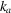 is zero. When the imaginary part of the anomalous scattering factor of Si is taken into account, 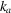 is 0.02. The blue thin and the black thick curves show MIFMD for 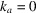 and 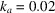 , respectively. Peaks indicated as M_1_ to M_7_ in the first zone and 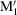 and 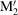 in the second zone are eventually attributed to MIFMD. When 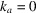 , the peak M_5_ is about twice higher than M_1_, and the height of 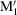 is comparable with that of M_1_. When 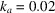 , all the peak heights are roughly a quarter of those for 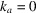 . The peak heights from M_1_ to M_7_ are not so much different, showing a similar trend of variations in the experiment. However, 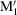 shows a similar peak height to M_1_ while 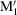 in Fig. 1 ▸(*a*) shows a much lower peak than M_1_. It is not possible to reproduce MIFMD observed in the experiment only by taking into account the effect of mean absorption.

**Figure 3:**
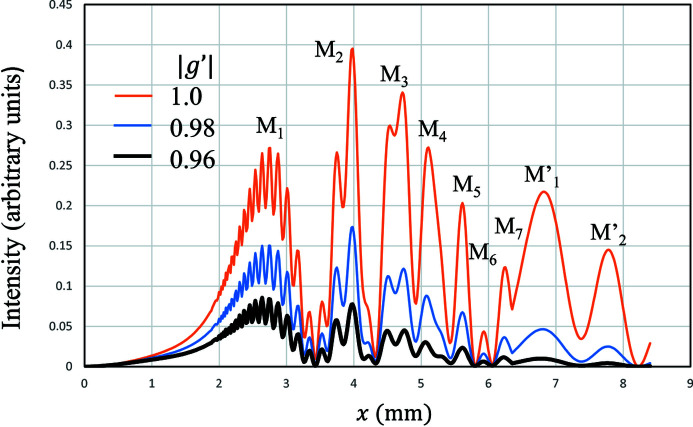
The calculated Si220 reflected intensities of MIFMD by taking the thermal vibration effect into account when β = 0.20 mm^−1^ and 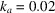 . The orange, blue and black lines show the intensities for 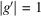 , 0.98 and 0.96, respectively.

The thermal vibration effect can be taken into account through 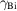 in equation (28), as it includes the term 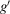 , which is given by

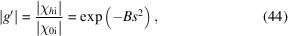

where *B* expresses the thermal vibration effect and 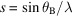 . The calculated MIFMD are shown in Fig. 3 ▸. The orange, blue and black lines show MIFMD for 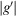 = 1.0, 0.98 and 0.96, respectively. When 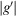 = 1.0, no thermal vibration is taken into account. When 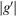 becomes small, the overall peak heights become small. When 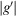 = 0.98, the peak height of M′_1_ is about 1/3 of M_1_. The peak height of M′_1_ becomes about 1/10 of M_1_ for 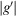 = 0.96. By comparing with the experimental results, we adopt 0.96 as the value of 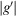 . The value of *B* corresponding to 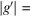 0.96 is 0.60 Å^2^, which is certainly larger than reported values such as 0.469 Å^2^ by Flensburg & Stewart (1999 ▸) and 0.4833 Å^2^ by Sang *et al.* (2010 ▸). The large value may come from an anisotropic or anharmonic vibrational effect. A further study is needed to confirm such a vibrational effect quantitatively.

### 4.2. Comparison of MIFMD with IFMD and IFMRB

**Figure 4:**
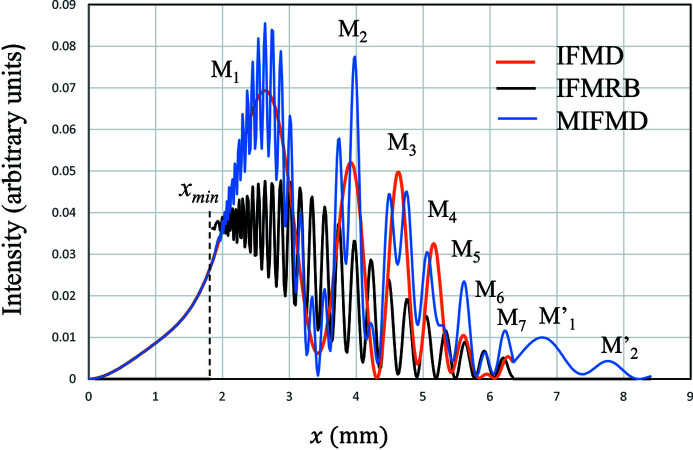
The calculated Si220 reflected intensities of IFMD (in orange), IFMRB (in black) and MIFMD (in blue) for β = 0.20 mm^−1^, 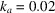 and 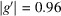 .

In Fig. 4 ▸ are shown the calculated intensities of IFMD (orange), IFMRB (black) and MIFMD (blue). The strain gradient β is assumed to be 0.20 mm^−1^. The intensity of IFMD shows a slow variation as a function of *x*. The width of a fringe and the interval between neighbouring fringes become small when *x* increases. The peak height in M*_n_* decreases as *n* (*x*) increases. The peak of M_6_ is extremely small compared with the neighbouring peaks. IFMRB starts to appear from 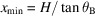 . The width of the fringe and the interval between neighbouring fringes become large as *x* increases. The intervals between neighbouring fringes of IFMRB are smaller than those of IFMD when 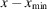 is small, but these two intervals are comparable when *x* becomes close to 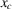 . For M_1_, MIFMD is approximately the sum of IFMD and IFMRB. For M_2_ and M_3_, two peaks appear in MIFMD by the influence of IFMRB. For M_3_, the peak of IFMD and the valley of IFMRB appear at the same *x* and the peak of MIFMD is lowered. For M_5_ to M_7_, the intervals of the fringes of IFMD and IFMRB are almost the same and the peak heights of MIFMD are enhanced more than twice those of IFMD.

### 4.3. Comparison of topographies

**Figure 5:**
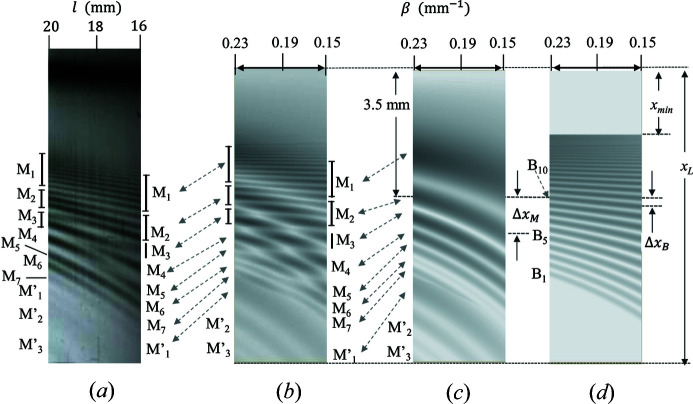
(*a*) The observed Si220 topography. (*b*), (*c*) and (*d*) show the calculated topographies of MIFMD, IFMD and IFMRB, respectively, for 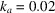 and 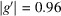 . Fringes in (*d*) are numbered as 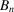 ( 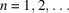 ) from 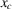 to 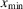 , as the phase 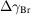 is zero at 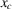 and becomes large as *x* decreases. The peak appears at a point *x* corresponding to 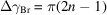 in equation (37).

Fig. 5 ▸ shows the measured topography (*a*), the calculated topographies of MIFMD (*b*), IFMD (*c*) and IFMRB (*d*). The abscissa is the distance *l* in (*a*) and the strain gradient β in (*b*)–(*d*). The correspondence between *l* and β is described in Section 2.

In Fig. 5 ▸(*a*), there are three dark contrasts of M_2_ on the left end (*l* = 20 mm). The uppermost dark contrast becomes the lowest fringe of dark contrast of M_1_ on the right end (*l* = 16 mm). There are two dark contrasts of M_3_ on the left end. The upper dark contrast is weaker than the lower one. The upper dark contrast is connected to the lowest dark contrast of M_2_ on the right end after showing weak contrast around *l* = 19 mm. The lower dark contrast of M_3_ on the left end becomes the dark contrast of M_3_ on the right end after showing weak contrast around *l* = 17 mm.

In Fig. 5 ▸(*b*), there are three dark contrasts of M_2_ on the left end. The dark uppermost contrast becomes weak around β = 0.19 mm^−1^, then it is connected to the lowest dark contrast of M_1_ on the right end. There are two dark contrasts of M_3_ on the left end. The upper contrast is weaker than the lower one. The contrast disappears around β = 0.21 mm^−1^, then becomes dark and the lowest dark contrast of M_2_ on the right end. The lowest dark contrast of M_3_ on the left end disappears around β = 0.17 mm^−1^, then becomes the dark contrast of M_3_ on the right end. Similar behaviours of fringe contrasts of the measured topography in Fig. 5 ▸(*a*) are obtained in the calculated MIFMD topography in (*b*).

In the calculated IFMD topography in Fig. 5 ▸(*c*), the fringe M_1_ shows a wide band of dark contrast. The higher-order fringes M_2_ to M_7_ show the narrower bands and the smaller interval between the neighbouring fringes than M_1_. In the calculated IFMRB topography in Fig. 5 ▸(*d*), the width of the dark contrasts becomes monotonously large and the distance between the neighbouring fringes becomes large when *x* increases from 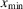 . Around *x* = 3.5 mm, the dark contrast of M_2_ appears at the left end in (*c*) and the dark contrast of B_10_ appears at the left end in (*d*). The shift of M_2_ is 1.2 mm from the left to the right end, while that of B_10_ is 0.3 mm. The shift of M_2_ is four times larger than that of B_10_. Similarly, the shift of M_7_ is larger than that of B_1_. The variations of interference fringes both in IFMD (*c*) and IFMRB (*d*) topographies are regular as a function of *x*. The irregular variations observed in experiment (*a*) are only reproduced in MIFMD topography (*b*). The irregular variations are caused by the different variations between IFMD and IFMRB as a function of *x* as well as β.

## 5. Discussion and conclusion

The irregular X-ray mirage interference fringes reported by Jongsukswat *et al.* (2013 ▸) were analysed using the dynamical theory of diffraction. It is necessary to take the absorption as well as thermal vibration effects into account. The absorption effect reduces the peak intensities of the high-order fringes in the first zone. The thermal vibration effect reduces mainly the peak intensities of fringes in the second zone. The calculated MIFMD reproduces the observed irregular modulation of the fringes. The origin of the modulation is attributed to the interference of two or more mirage diffracted beams with a beam reflected from the back surface.

**Figure 6:**
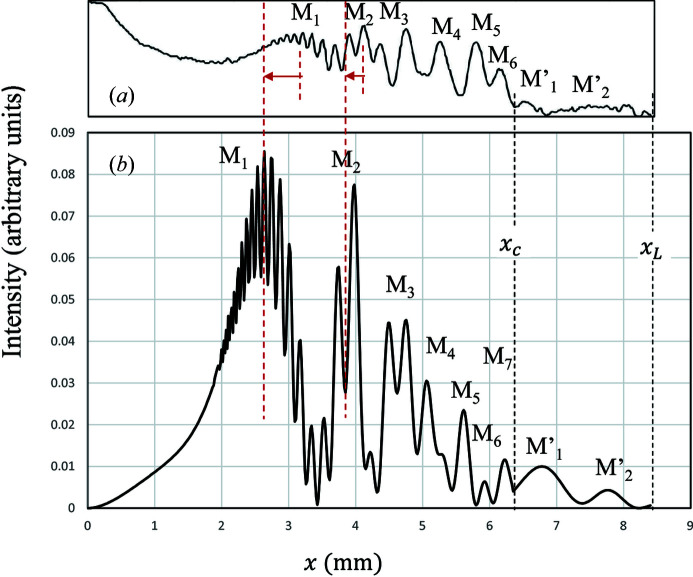
(*a*) The intensity profile of the observed Si220 topography along the white dashed line in Fig. 1 ▸(*a*). (*b*) The corresponding calculated intensity profile of MIFMD for β = 0.20 mm^−1^, 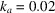 and 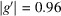 .

There are still two points that are unclear. (i) Fig. 6 ▸ shows (*a*) the line profile of the measured fringes along the dashed line in Fig. 1 ▸(*a*) and (*b*) that of the calculated reflected intensities of MIFMD. The peak positions of M_1_ and M_2_ in the calculated profile appear at *x* closer to the incident point than in the measured one. One of the possible reasons is the dependence of the strain gradient (β) on the distance (*x*), which is difficult to estimate by using the deflection theory. More precise analysis of the MIFMD is necessary in future work. (ii) The other point is related to IFLSD observed in Fig. 1 ▸(*a*). The intensities of IFLSD are much higher than those of MIFMD in the second zone. Hirano *et al.* (2008 ▸, 2009*a* ▸,*b* ▸) have observed IFLSD from a plane-parallel crystal without distortion and pointed out that the fringes are caused by the interference between the beam directly reaching the lateral surface and the beam reflected once from the back surface. In the present geometry of a bent crystal, there is no beam reaching directly the lateral surface as shown in Fig. 1 ▸(*c*). It is necessary to apply the dynamical theory of diffraction for a distorted crystal for analysing the IFLSD and the strain gradient of the crystal. As an application, the X-ray beams of IFLSD can be used as an X-ray waveguide, since they propagate quite a long distance from the incident point to the exit point. Fukamachi *et al.* (2011*a* ▸) carried out an experiment on an X-ray diffractometer by using X-rays of IFLSD from a plane-parallel crystal as a waveguide and beam splitter. Constructive interference between many beams is derived in the present analysis of MIFMD, which should be useful for developing an X-ray waveguide using IFLSD from a bent crystal.
